# Development and validation of a model integrating clinical and metabolomic markers for gestational diabetes mellitus prediction

**DOI:** 10.3389/fmed.2026.1806848

**Published:** 2026-06-17

**Authors:** Guixi Wang, Wenfeng Zhang

**Affiliations:** 1 Department of Obstetrics, Jinan Central Hospital, Jinan, China; 2 Department of Obstetrics and Gynecology, The First Affiliated Hospital of Shandong First Medical University and Shandong Provincial Qianfoshan Hospital, Jinan, China

**Keywords:** early diagnosis, gestational diabetes mellitus, machine learning, metabolomics, nomogram, prediction model

## Abstract

**Background:**

Gestational diabetes mellitus (GDM) is a prevalent pregnancy complication. Current diagnostic approaches are inherently retrospective, necessitating the development of effective early prediction models for timely intervention and improved outcomes.

**Objective:**

This study aimed to develop and validate a prediction model for GDM risk by integrating first-trimester clinical and metabolomic indicators.

**Methods:**

A retrospective cohort study was conducted involving 342 singleton pregnancies that received routine antenatal care and underwent mid-pregnancy oral glucose tolerance tests (OGTT) from January 2022 to December 2024. Participants were randomly allocated to a training set (n = 239) and a validation set (*n* = 103) in a 7:3 ratio. Core predictors were identified through a univariate analysis, LASSO regression, and subsequent multivariable logistic regression. Four machine learning models—Random Forest, Support Vector Machine (SVM), Gradient Boosting Machine, and Logistic Regression—were constructed and compared. Performance was evaluated by the area under the curve (AUC), calibration curves, and decision curve analysis. Model interpretability was assessed using SHapley Additive exPlanations (SHAP) values.

**Results:**

A multivariable analysis identified seven independent predictors: pre-pregnancy BMI, first-trimester fasting plasma glucose, triglycerides, C-reactive protein, and the branched-chain amino acid score (risk factors), as well as pregnancy-associated plasma protein-A and 1,5-anhydroglucitol (protective factors). In the validation set, the SVM model achieved optimal performance with an AUC of 0.861 (95% confidence interval (CI): 0.772–0.949). Calibration and decision curve analyses demonstrated good agreement between predicted and observed risks and affirmed clinical utility across a wide threshold probability range.

**Conclusion:**

A prediction model integrating first-trimester clinical and metabolomic markers was successfully developed and validated. The model demonstrates favorable predictive accuracy and clinical applicability, offering potential as an auxiliary tool for early risk stratification and personalized GDM management. Future multi-center external validation is warranted to confirm generalizability.

## Introduction

Gestational diabetes mellitus (GDM) is a common metabolic complication of pregnancy. It is closely associated with an increased risk of adverse short- and long-term health outcomes for both mothers and offspring, including macrosomia, higher rates of cesarean delivery, and offspring metabolic abnormalities (1). Currently, the diagnosis of GDM primarily relies on an oral glucose tolerance test (OGTT) performed at 24–28 weeks of gestation (2). This diagnostic paradigm exhibits a clear time lag, which is detrimental to initiating early prevention and intervention (3). Consequently, identifying women at high risk for GDM during early pregnancy or even before conception holds significant clinical importance for implementing targeted management and improving prognosis.

Research indicates that the pathogenesis of GDM involves multiple pathophysiological mechanisms, including insulin resistance, chronic low-grade inflammation, and dyslipidemia (4). The known clinical risk factors encompass advanced maternal age, high pre-pregnancy body mass index (BMI), and a family history of diabetes (5). In recent years, metabolomics studies have revealed that altered levels of specific early-pregnancy metabolites (e.g., branched-chain amino acids and acylcarnitines) may be associated with subsequent GDM development, offering novel potential biomarkers for early prediction (6). However, the predictive power of individual clinical indicators or metabolites is limited. A key research focus, therefore, is how to effectively integrate easily obtainable early-pregnancy clinical characteristics with emerging metabolomic information to construct an efficient and practical early risk prediction tool.

Machine learning algorithms are capable of handling complex, high-dimensional data and capturing non-linear interactions among variables, demonstrating advantages in developing disease risk prediction models. This study aims to retrospectively collect multi-dimensional data during early pregnancy, including demographic, clinical, and targeted metabolomic data. By using integrated statistical methods and machine learning techniques, we intend to develop and validate a model for predicting the risk of GDM onset.

## Materials and methods

### Study participants

Pregnant women who attended our hospital’s obstetrics clinic for regular prenatal care and completed a mid-pregnancy OGTT between January 2022 and December 2024 were retrospectively enrolled. First, a sample size estimation was performed. Based on the reported GDM prevalence of approximately 25% in previous literature and adhering to the rule of at least 10 events per predictor variable (EPV) for predictive model development (7), a minimum of 60–70 GDM events was required for 6–7 candidate predictors. Given a GDM prevalence of 25%, the corresponding total sample size would be at least 240–280 participants. To further account for an estimated 10% prevalence of incomplete data, the final target sample size was set to approximately 264–308 participants. Ultimately, 342 pregnant women were included, comprising a training set (n = 239) and a validation set (n = 103), which satisfied the sample size requirements for model construction and internal validation. The 7:3 split was selected as the gold-standard proportion for clinical prediction model development: It provides a sufficient sample size for model training to capture complex variable relationships while retaining an adequate independent validation set to test model stability. This ratio also satisfies the key requirement of at least 10 outcome events per candidate predictor for reliable GDM prediction modeling.

The inclusion criteria were as follows: (1) singleton pregnancy; (2) establishment of a prenatal healthcare record at our hospital and completion of the first prenatal visit before 12 weeks of gestation, with baseline data and blood samples collected; (3) completion of the standard 75-g OGTT at our hospital at 24–28 weeks of gestation; and (4) availability of complete clinical data. The exclusion criteria were as follows: (1) pre-existing diabetes or impaired glucose tolerance diagnosed before pregnancy; (2) severe comorbidities affecting major organs (the heart, liver, and kidney); (3) endocrine disorders influencing glucose metabolism (e.g., hyperthyroidism and Cushing’s syndrome); (4) use of medications affecting glucose metabolism during pregnancy (e.g., corticosteroids); and (5) missing baseline or OGTT follow-up data.

### Data collection

Data for all included participants were collected from the hospital’s electronic medical record system and the perinatal healthcare system.

At baseline (early pregnancy and <12 weeks of gestation), we collected comprehensive demographic and medical history information, including maternal age, height, pre-pregnancy weight (from which pre-pregnancy body mass index [BMI] was calculated), parity, family history of diabetes, personal history of polycystic ovary syndrome, and history of chronic hypertension. Additionally, a panel of clinical indicators was assessed, encompassing early-pregnancy systolic and diastolic blood pressure, fasting plasma glucose, lipid profiles (e.g., triglycerides and high-density lipoprotein cholesterol), liver and renal function parameters (including serum creatinine), C-reactive protein, and pregnancy-associated plasma protein-A. For metabolomic profiling, fasting serum samples obtained in early pregnancy were analyzed using targeted metabolomics approaches; the measured and derived parameters included a branched-chain amino acid score (incorporating valine, leucine, and isoleucine), as well as levels of tyrosine, 1,5-anhydroglucitol, acylcarnitine C3, and trimethylamine N-oxide. All metabolomic data underwent standardized preprocessing to ensure consistency.

For the diagnostic assessment conducted during mid-pregnancy (24–28 weeks of gestation), we recorded plasma glucose concentrations at fasting, 1 h, and 2 h following a standard 75-g oral glucose tolerance test (OGTT), along with mid-pregnancy levels of glycated hemoglobin (HbA1c).

### Outcome definition

GDM diagnosis was based on the International Association of Diabetes and Pregnancy Study Groups (IADPSG) criteria using the 75-g OGTT results at 24–28 weeks of gestation (8). GDM was diagnosed if any of the following thresholds were met: fasting plasma glucose ≥5.1 mmol/L, or 1-h post-load glucose ≥10.0 mmol/L, or 2-h post-load glucose ≥8.5 mmol/L. Accordingly, all participants were categorized into the GDM and non-GDM groups. All OGTT measurements were performed by the hospital laboratory following a standardized protocol.

### Statistical analysis

Statistical analysis was conducted using SPSS 26.0 and Python 3.9. Continuous variables conforming to a normal distribution are presented as mean±standard deviation (x̄±s) and were compared between groups using the independent samples *t*-test. Non-normally distributed continuous variables are presented as median (interquartile range) and were compared using the Mann–Whitney U-test. Categorical variables are presented as numbers (percentages) and were compared using the chi-squared test.

The total cohort was randomly classified into a training set and a validation set in a 7:3 ratio. We used a stepwise, transparent variable screening process in the training set: A univariate analysis was used to preliminarily screen indicators with a *p*-value of <0.05; Least Absolute Shrinkage and Selection Operator (LASSO) regression was applied for further feature selection to reduce overfitting; a multivariable logistic regression analysis was conducted to identify independent GDM predictors.

To identify the optimal predictive algorithm, to verify the stability of core predictors, and to capture both linear and non-linear relationships between variables and GDM risk, we then built four machine learning models [random forest (RF), support vector machine (SVM), gradient boosting machine (GBM), and logistic regression (LR)] using Python’s scikit-learn library. Hyperparameters were optimized via 10-fold cross-validation in the training set. The discriminatory performance of the models was evaluated using the area under the receiver operating characteristic (ROC) curve (AUC) and subsequently validated on the independent validation set. Calibration curves were used to assess the agreement between predicted probabilities and the actual risk. Decision curve analysis was applied to evaluate the clinical utility of the models. Finally, the SHapley Additive exPlanations (SHAP) framework was utilized for interpretability analysis of the optimal machine learning model. A *p*-value of < 0.05 was considered statistically significant.

## Results

### Comparison of baseline characteristics between the training and validation sets

This study included 342 pregnant women who completed early-pregnancy assessments and a mid-pregnancy OGTT. They were randomly divided into a training set (n = 239) and a validation set (n = 103) in a 7:3 ratio. Comparisons of baseline characteristics between the training and validation sets—including demographics and medical history (e.g., age, pre-pregnancy BMI, and family history of diabetes), early-pregnancy clinical indicators (e.g., blood pressure, fasting plasma glucose, lipid profile, and C-reactive protein), early-pregnancy metabolomic indicators (e.g., branched-chain amino acids, specific acylcarnitines, and trimethylamine N-oxide), and mid-pregnancy diagnostic-related indicators (OGTT glucose values and glycated hemoglobin)—showed no statistically significant differences (*p* > 0.05). These findings indicate a balanced split between the training and validation sets, with comparable baseline characteristics suitable for analysis (Table 1).

**Table 1:** Comparison of baseline characteristics and pregnancy indicators between pregnant women in the training and validation sets.

Variable	Training set (*n* = 239)	Validation set (*n* = 103)	*t/χ^2^*	*p*
Age (years)	30.69 ± 4.31	30.81 ± 4.14	0.239	0.811
Pre-pregnancy BMI (kg/m^2^)	23.9 ± 3.6	24.0 ± 3.5	0.326	0.745
Family history of diabetes (yes/no)	65 (27.2%)/174 (72.8%)	30 (29.1%)/73 (70.9%)	0.134	0.715
History of PCOS (yes/no)	26 (10.9%)/213 (89.1%)	12 (11.7%)/91 (88.3%)	0.043	0.835
History of chronic hypertension (yes/no)	14 (5.9%)/225 (94.1%)	7 (6.8%)/96 (93.2%)	0.111	0.741
SBP in first trimester (mmHg)	115.53 ± 10.24	115.81 ± 9.87	0.235	0.815
DBP in first trimester (mmHg)	72.11 ± 8.41	71.82 ± 8.55	0.291	0.771
FBG in first trimester (mmol/L)	4.64 ± 0.41	4.61 ± 0.38	0.643	0.521
Triglycerides in first trimester (mmol/L)	1.59 ± 0.51	1.63 ± 0.48	0.677	0.499
HDL-C in first trimester (mmol/L)	1.71 ± 0.34	1.68 ± 0.36	0.735	0.463
CRP in first trimester (mg/L)	2.05 ± 1.11	2.12 ± 1.08	0.539	0.591
PAPP-A (MoM)	1.07 ± 0.32	1.05 ± 0.34	0.521	0.603
Total branched-chain amino acid score (standardized)	0.03 ± 1.01	0.05 ± 0.99	0.169	0.866
Tyrosine (μmol/L)	48.51 ± 9.13	47.76 ± 9.03	0.699	0.485
1,5-Anhydroglucitol (μg/mL)	22.01 ± 6.54	21.63 ± 6.39	0.496	0.621
Acylcarnitine C3 (μmol/L)	0.28 ± 0.09	0.27 ± 0.08	0.974	0.331
Trimethylamine N-oxide (μmol/L)	3.12 ± 1.21	3.05 ± 1.19	0.493	0.622
Mid-trimester OGTT-fasting glucose (mmol/L)	4.91 ± 0.58	4.85 ± 0.57	0.882	0.378
Mid-trimester OGTT-1-h glucose (mmol/L)	8.71 ± 2.03	8.59 ± 1.91	0.511	0.611
Mid-trimester OGTT-2-h glucose (mmol/L)	7.62 ± 1.74	7.53 ± 1.61	0.449	0.654
Mid-trimester HbA1c (%)	5.16 ± 0.35	5.14 ± 0.36	0.481	0.631

### Univariate analysis of early-pregnancy predictors for GDM in the training cohort

Within the training cohort of 239 pregnant women, 60 were diagnosed with GDM via OGTT in the mid-trimester (GDM group), and 179 were non-GDM controls (non-GDM group). A univariate analysis revealed statistically significant differences (*p* < 0.05) between the GDM and non-GDM groups in pre-pregnancy BMI, first-trimester FBG, first-trimester TG, first-trimester CRP, placenta function-related marker (PAPP)-A, total branched-chain amino acid (BCAA) score, and 1,5-anhydroglucitol levels (Table 2).

**Table 2:** Comparison of baseline characteristics by univariate analysis in the training set.

Variable	Non-GDM group (*n* = 179)	GDM group (*n* = 60)	*t*/*χ*^2^	*p*
Age (years)	30.51 ± 3.91	31.32 ± 4.84	1.305	0.193
Pre-pregnancy BMI (kg/m^2^)	23.21 ± 3.11	26.09 ± 4.01	5.752	0.001
Family history of diabetes (yes/no)	44 (24.6%)/135 (75.4%)	21 (35.0%)/39 (65.0%)	2.463	0.117
History of PCOS (yes/no)	17 (9.5%)/162 (90.5%)	9 (15.0%)/51 (85.0%)	1.404	0.236
History of chronic hypertension (yes/no)	8 (4.5%)/171 (95.5%)	6 (10.0%)/54 (90.0%)	1.591	0.207
SBP in first trimester (mmHg)	114.11 ± 9.52	116.52 ± 11.01	1.629	0.104
DBP in first trimester (mmHg)	71.41 ± 8.03	73.09 ± 9.21	1.351	0.178
FBG in first trimester (mmol/L)	4.56 ± 0.36	4.90 ± 0.41	6.109	0.001
Triglycerides in first trimester (mmol/L)	1.48 ± 0.46	1.92 ± 0.53	6.166	0.001
HDL-C in first trimester (mmol/L)	1.70 ± 0.35	1.73 ± 0.29	0.598	0.550
CRP in first trimester (mg/L)	1.82 ± 0.98	2.75 ± 1.15	6.082	0.001
PAPP-A (MoM)	1.13 ± 0.33	0.92 ± 0.26	4.483	0.001
Total branched-chain amino acid score (standardized)	−0.15 ± 0.94	0.62 ± 1.05	5.329	0.001
Tyrosine (μmol/L)	47.91 ± 8.52	50.11 ± 9.32	1.691	0.092
1,5-Anhydroglucitol (μg/mL)	23.51 ± 6.41	18.25 ± 5.52	5.687	0.001
Acylcarnitine C3 (μmol/L)	0.27 ± 0.08	0.29 ± 0.11	1.516	0.131
Trimethylamine N-oxide (μmol/L)	2.95 ± 1.12	3.17 ± 1.28	1.269	0.206
Mid-trimester OGTT-fasting glucose (mmol/L)	4.90 ± 0.41	4.93 ± 0.39	0.496	0.620
Mid-trimester OGTT-1-h glucose (mmol/L)	8.69 ± 1.51	8.75 ± 1.52	0.266	0.791
Mid-trimester OGTT-2-h glucose (mmol/L)	7.60 ± 1.39	7.66 ± 1.35	0.291	0.771
Mid-trimester HbA1c (%)	5.15 ± 0.31	5.18 ± 0.35	0.627	0.531

### Screening via LASSO regression and the multivariate logistic regression analysis for GDM predictors in the training cohort

This study defined the diagnosis of GDM in the mid-trimester as the dependent variable (Y), where Y = 1 represents the GDM group and Y = 0 represents the non-GDM group. All seven first-trimester indicators showing statistical significance in the univariate analysis were included for variable selection via LASSO regression (Supplementary Table 1). The optimal variables were selected using 10-fold cross-validation and the *λ*-1se criterion. Ultimately, all seven predictive variables were retained and incorporated into the multivariate logistic regression model.

**Figure 1:**
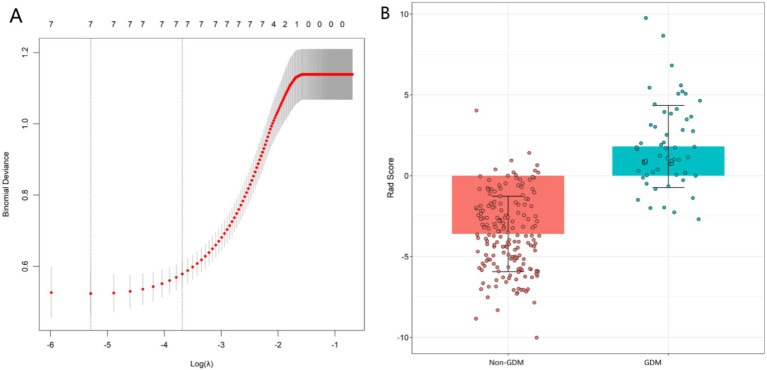
LASSO regression plot **(A)** and LASSO core difference comparison plot **(B)**.

The results from the multivariate logistic regression analysis (Table 3) indicated that pre-pregnancy BMI, first-trimester FBG, TG, CRP, and the total BCAA score were independent risk factors for GDM onset (OR>1, *p* < 0.05). In contrast, PAPP-A and 1,5-anhydroglucitol were independent protective factors (OR<1, *p* < 0.05; Figure 1).

**Table 3:** Multivariate logistic regression analysis of GDM predictors in the training cohort.

Variable	*β*	SE	Wald	*p*	OR	95% CI
Pre-pregnancy BMI	0.292	0.082	12.737	0.001	1.339	1.141 ~ 1.572
FBG in first trimester	0.736	0.270	7.438	0.001	2.088	1.228 ~ 3.543
Triglycerides in first trimester	0.853	0.217	15.445	0.001	2.346	1.533 ~ 2.590
CRP in first trimester	1.046	0.293	13.661	0.001	2.847	1.635 ~ 4.959
PAPP-A	−1.326	0.542	6.005	0.001	0.265	0.092 ~ 0.763
Total branched-chain amino acid score	1.217	0.322	14.298	0.001	3.413	1.924 ~ 6.047
1,5-Anhydroglucitol	−0.105	0.039	7.454	0.006	0.901	0.834 ~ 0.971

### Performance evaluation of machine learning models

**Figure 2:**
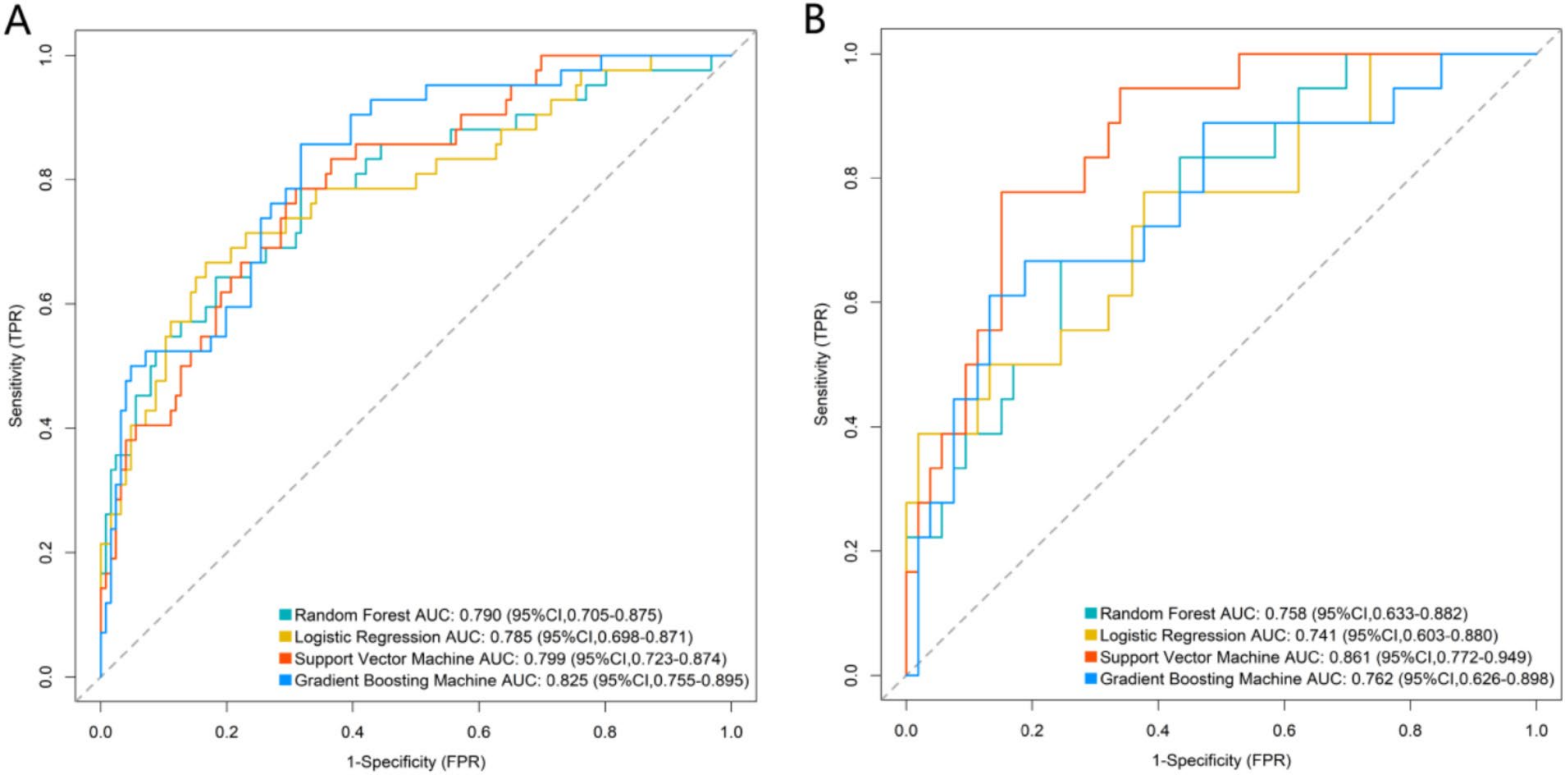
ROC curve analysis of the prediction model in the training **(A)** and validation **(B)** sets.

**Figure 3:**
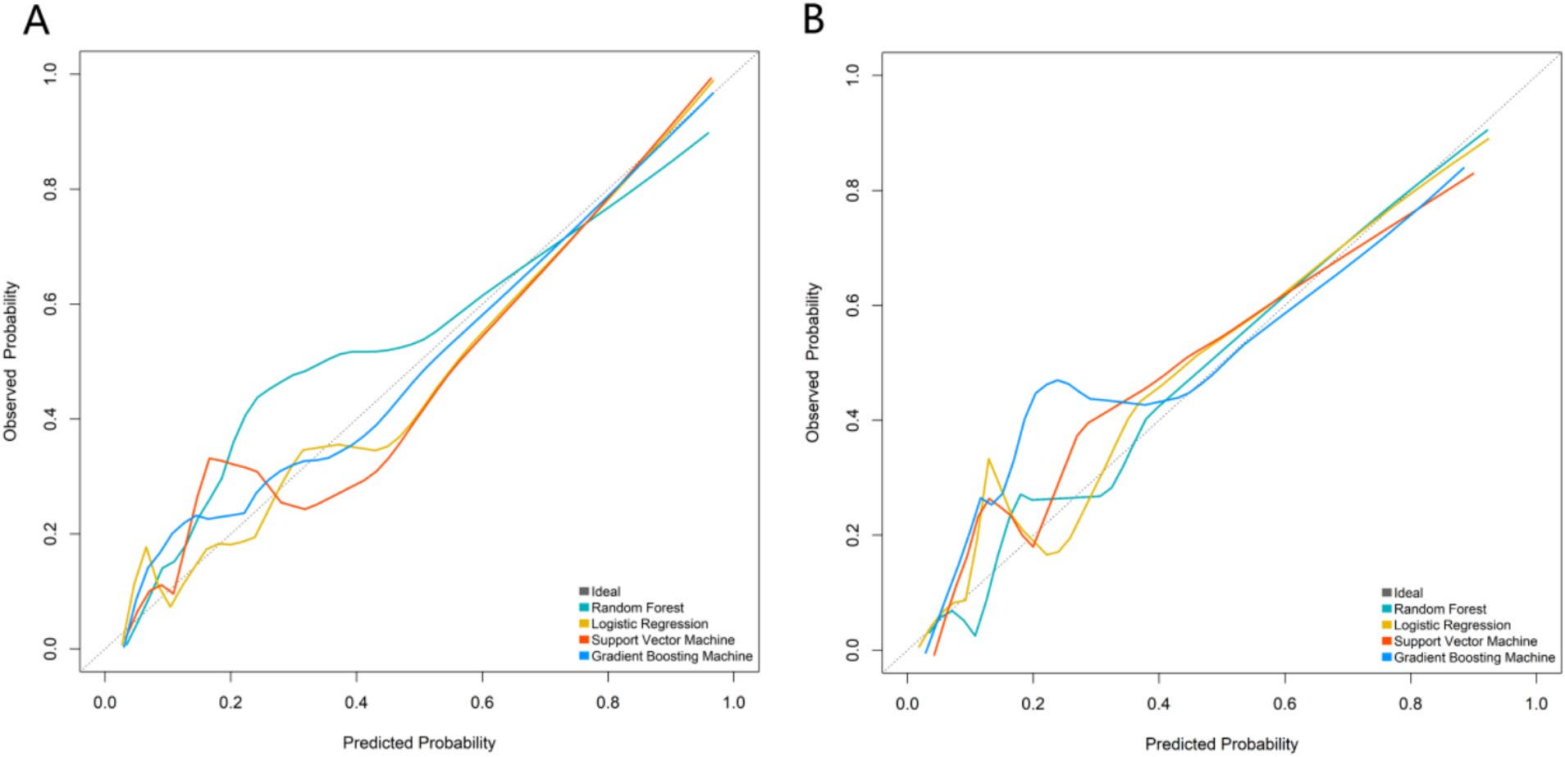
Curve analysis of the prediction model in the training **(A)** and validation **(B)** sets.

**Figure 4:**
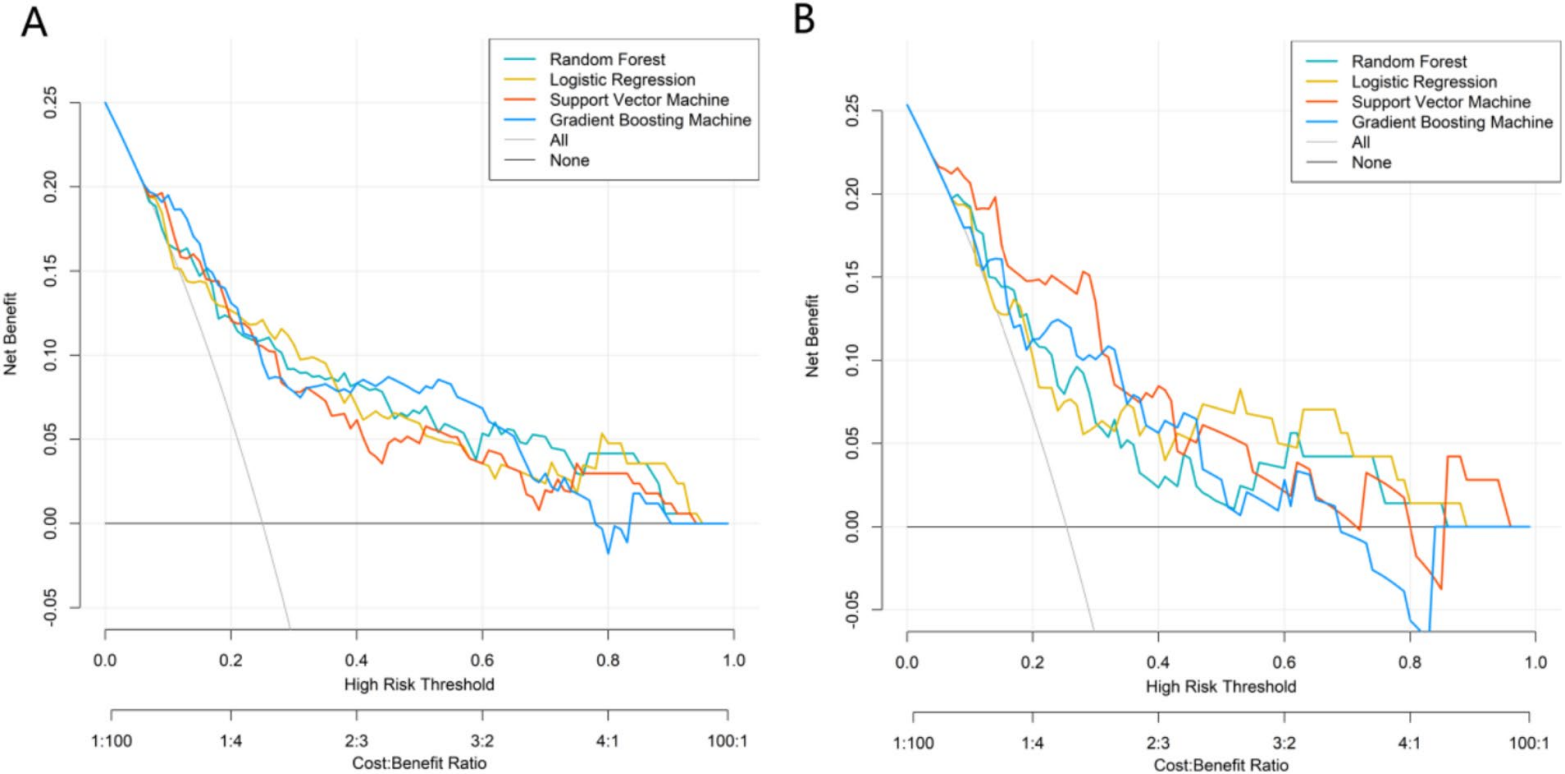
Clinical decision curve analysis of the prediction model in the training **(A)** and validation **(B)** sets.

Based on the seven key early-pregnancy predictors identified by the multivariate logistic regression analysis, this study further constructed four machine learning prediction models, RF, LR, SVM, and GBM, to systematically evaluate and optimize early GDM prediction performance. By comparing model performance on the training and validation sets, all models demonstrated good discriminative ability. Among them, the SVM model achieved the highest AUC of 0.861 (95% CI: 0.772–0.949) in the validation set. The validation set AUCs for the RF, GBM, and LR models were 0.758, 0.762, and 0.741, respectively (Figure 2). The comparable performance across models indicates the robustness of the prediction framework built upon multi-dimensional first-trimester indicators. The calibration curve analysis (Figure 3) showed good agreement between predicted probabilities and actual risk for all models. The decision curve analysis further confirmed (Figure 4) that, across a wide range of threshold probabilities, applying these prediction models provided higher clinical net benefit compared to the “treat-all” or “treat-none” strategies, with the RF model yielding the highest comprehensive net benefit. In summary, the machine learning prediction models developed in this study, based on first-trimester clinical and metabolomic indicators, possess good predictive accuracy, calibration, and clinical utility, offering a reliable quantitative tool for early GDM risk stratification and personalized intervention.

### Interpretability assessment of model predictions

**Figure 5:**
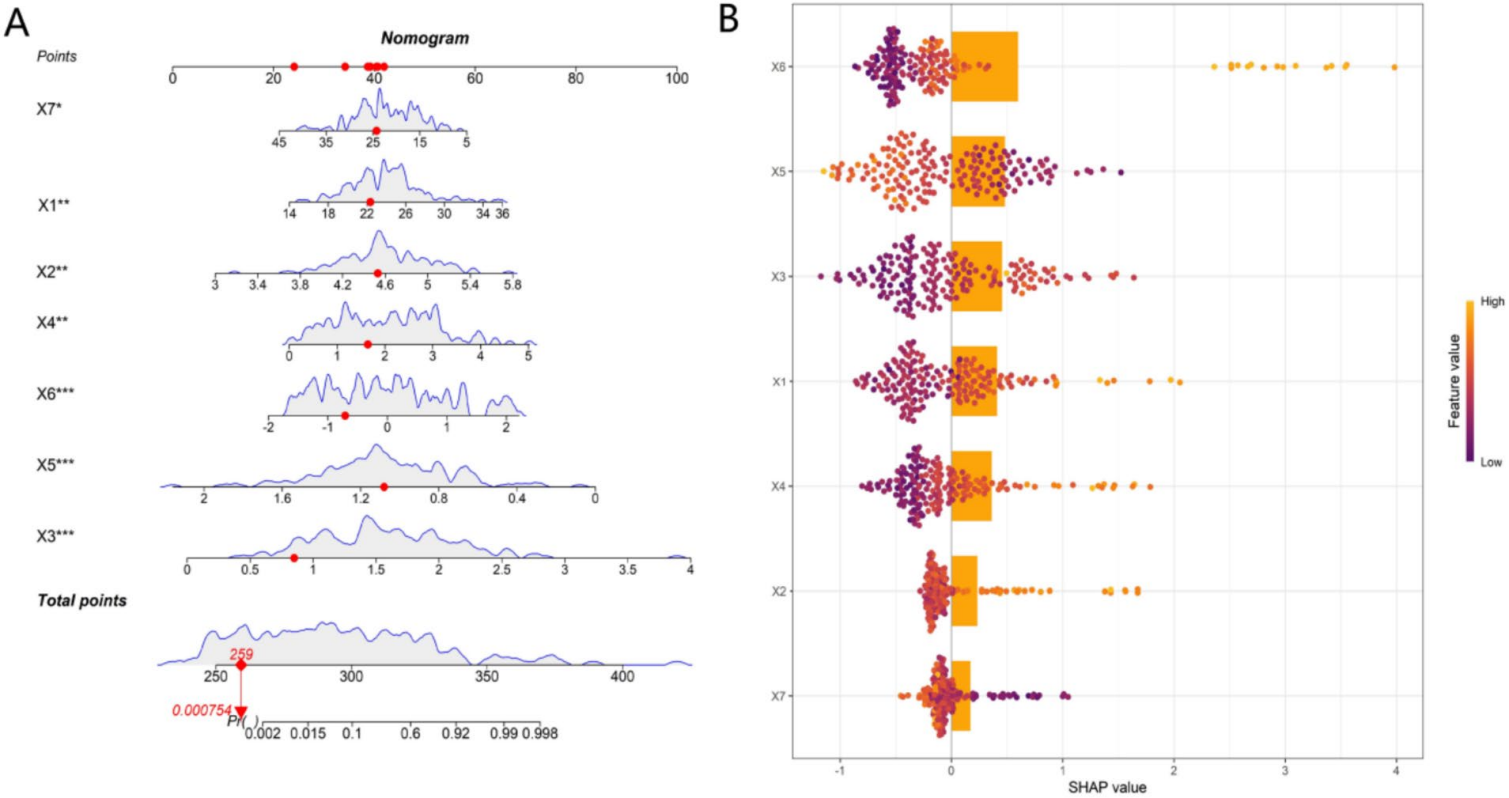
Model interpretability analysis. **(A)** Nomogram and **(B)** SHAP feature importance plot. Note: X1: pre-pregnancy BMI; X2: first-trimester FBG; X3: first-trimester TG; X4: first-trimester CRP; X5: PAPP-A; X6: total BCAA score; and X7: 1,5-anhydroglucitol.

Based on the seven core first-trimester predictors (pre-pregnancy BMI, FBG, TG, CRP, PAPP-A, total BCAA score, and 1,5-anhydroglucitol) selected via the optimal SVM model, this study developed a visual prediction tool (nomogram, Figure 5A) to intuitively display the contribution of each indicator to GDM risk. The model confirmed that first-trimester FBG, TG, CRP, and the total BCAA score were independent risk factors for GDM, where increased values significantly elevated the disease risk. Conversely, PAPP-A and 1,5-anhydroglucitol were protective factors.

To elucidate the model’s decision-making mechanism, SHAP analysis was used to quantify the global importance of each feature (Figure 5B). The results showed that the total BCAA score (highest SHAP value) contributed most prominently to the model’s predictions, indicating that it provided crucial incremental predictive information beyond traditional clinical indicators. This was followed by first-trimester FBG and CRP, whose importance was also confirmed. Notably, PAPP-A, as a strong protective factor, exhibited significant negative SHAP values, corroborating its inverse relationship with GDM risk.

## Discussion

Through a rigorous selection process, this study identified seven key predictive indicators: pre-pregnancy BMI, first-trimester FPG, TG, CRP, PAPP-A, a BCAA composite score, and 1,5-anhydroglucitol. The multivariable analysis demonstrated that a majority of these indicators were independently associated with GDM risk. Among them, elevated first-trimester FPG showed the most definitive association with increased GDM risk, consistent with conclusions from previous research (9). Notably, within the first-trimester cohort of this study, the mean FPG remained within the normal range. However, even subtle elevations within this range indicated diminished glucoregulatory capacity, reinforcing the clinical perspective that glucose levels approaching the upper limit of normal should be considered an important risk signal (10). The confirmed predictive value of pre-pregnancy BMI, an easily obtainable metric, underscores the fundamental role of pre-conception weight management in the primary prevention of GDM (11). Furthermore, the independent predictive role of first-trimester triglycerides suggests that early-pregnancy lipid metabolism status warrants attention alongside glucose metabolism as its dysregulation may represent an early concomitant manifestation of insulin resistance (12).

The significant contribution of the BCAA composite score, a representative metabolomic indicator incorporated into our model, merits focused discussion (13). Existing research suggests that elevated BCAA levels may be associated with the early development of insulin resistance. Our findings provide supportive evidence for this association. In particular, BCAAs (e.g., leucine, isoleucine, and valine) are not only substrates for protein synthesis; their metabolites can interfere with insulin signaling pathways, thereby impairing glucose uptake in tissues such as skeletal muscle (14). Our model revealed that the BCAA composite score remained a strong predictor of GDM even after adjusting for traditional glycemic and lipid measures. This finding provides novel corroboration from a metabolomic perspective for the early pathogenesis of GDM, implying that subtle alterations in metabolite profiles may harbor predictive information earlier than conventional clinical indicators can detect (15). The protective effect associated with 1,5-anhydroglucitol, a sensitive negative indicator reflecting average glycemia over the preceding 1–2 weeks, is also consistent with this pathophysiological logic. A decreased level of 1,5-anhydroglucitol indirectly suggests the possible presence of minor hyperglycemic excursions in the first trimester that are not captured by routine FPG testing (16).

In addition, an inflammatory marker (CRP) and a placenta function-related marker (PAPP-A) were included in the final model. Elevated CRP levels reflect low-grade inflammation, which can exacerbate insulin resistance through mechanisms such as interference with insulin receptor substrate signaling (17). PAPP-A is predominantly secreted by placental trophoblasts, and its reduced levels may be associated with early placental development or perfusion, potentially affecting subsequent metabolic adaptation in pregnancy (18). Both markers have been previously reported to correlate with GDM risk. Their inclusion in our model suggests that the onset of GDM is not attributable to a single metabolic pathway abnormality. Instead, it likely involves the early, subtle interplay among multiple pathophysiological processes, including metabolism, inflammation, and placental function (19). By integrating these three distinct biomarker dimensions, our model constructs a more comprehensive framework for first-trimester risk assessment.

This study was designed as a retrospective observational cohort study. All participants received only routine standard antenatal care following the clinical norms of our hospital throughout pregnancy. No targeted dietary, lifestyle, exercise, or pharmacological interventions to reduce the risk of gestational diabetes mellitus (GDM) were implemented in either the training set or the validation set. This pure observational design was adopted to avoid the confounding effects of preventive interventions on the natural occurrence of GDM, ensuring the authenticity of outcome data for model development and validation.

From a methodological standpoint, this study used a stepwise analytical strategy from feature selection to model construction. First, LASSO regression was used to screen key predictors and reduce overfitting. Second, we evaluated multiple machine learning models simultaneously, which is a rigorous design to identify the optimal algorithm and confirm the stability of the feature set. The consistent predictive performance across different models further verified the reliability of the seven core indicators. The application and comparison of various machine learning algorithms were conducted not only to seek optimal predictive performance but also, more importantly, to indirectly validate the reliability of the selected feature combination through their consistent performance across different algorithms on the validation set.

Regarding the choice of a specific model for ultimate clinical implementation, while the SVM model achieved the highest AUC (0.861) in the validation set, the differences in predictive accuracy among the four models were relatively modest. In practice, model selection may depend on factors beyond raw efficacy. For instance, LR offers the advantages of simplicity, ease of interpretation, and seamless integration into electronic health record systems as a risk-scoring formula. RF and GBM, though potentially more computationally intensive, can capture complex non-linear interactions and may be preferred in settings with dedicated data science support. SVM strikes a balance between performance and interpretability, particularly when using linear kernels. Given that our primary goal is to develop a practical tool for early GDM risk stratification, we suggest that the final model choice should be guided by the specific clinical context, user accessibility, and computational resources available at the implementing institution. A simplified nomogram based on the SVM-derived coefficients or a web-based calculator could enhance user-friendliness and promote clinical adoption. Future prospective implementation studies may help clarify which model offers the best trade-off between accuracy and usability in real-world antenatal care settings. Notably, the application of SHAP analysis facilitated an intuitive understanding of the contribution direction and relative magnitude of each feature to the model’s predictions. This approach provides visual and quantitative interpretation of the decision-making process in complex machine learning models, which are traditionally viewed as “black boxes.” For instance, SHAP analysis can visually demonstrate that, for a specific high-risk individual, an elevated BCAA level is the primary factor driving the model’s high-risk prediction. This enhances, to a degree, the clinical interpretability of complex models, rendering the predictions more persuasive and potentially indicating directions for targeted personalized interventions (20).

Early identification of GDM is crucial for implementing timely interventions and improving pregnancy outcomes. The traditional diagnostic approach, which relies on the OGTT performed at 24–28 weeks of gestation, exhibits inherent limitations due to its delayed timing (20). In clinical practice, patients identified as being at very high risk during the first trimester could undergo OGTT as early as 13 weeks of gestation for earlier diagnosis. This retrospective observational cohort study, without targeted GDM preventive interventions, aimed to develop an early prediction model for GDM based on clinically accessible indicators and metabolomic profiles available in the first trimester, with the goal of providing a reference for clinical risk stratification and guiding earlier selective screening.

Regarding the practical implementation of our prediction model in clinical settings, we envision a straightforward workflow. During the first-trimester prenatal visit (before 12 weeks of gestation), a pregnant woman would undergo routine blood sampling. Our model requires seven predictors: pre-pregnancy BMI, first-trimester fasting plasma glucose, triglycerides, C-reactive protein, PAPP-A, total branched-chain amino acid (BCAA) score, and 1,5-anhydroglucitol. While the first five are already part of standard panels in many centers, the BCAA score and 1,5-anhydroglucitol would require targeted metabolomic assays, which can be performed on the same blood sample if resources permit. Once these values are obtained, they can be entered into a simple interface—such as a nomogram (Figure 5A), a spreadsheet-based calculator, or an automated module integrated into the electronic health record system. The model then outputs an estimated GDM risk probability. Based on a prespecified risk threshold (e.g., 0.3 or 0.5), patients can be stratified into low-, moderate-, and high-risk categories. High-risk individuals may be offered early OGTT screening (e.g., at 13–16 weeks of gestation) or receive intensified lifestyle counseling, whereas low-risk individuals can continue routine antenatal care.

Regarding the training requirements for medical professionals, we emphasize that no specialized machine learning expertise is needed. Obstetricians, family physicians, midwives, and other prenatal care providers would only require a brief orientation (e.g., a 15-min tutorial or a one-page instruction sheet) to understand how to access the calculator, interpret the risk scores, and follow the corresponding clinical algorithms. Laboratory personnel would follow standard quality control procedures for metabolomic assays, which do not require additional clinical training. For settings where metabolomic testing is not readily available, a reduced model using only clinical variables could be considered in future studies as a more accessible alternative, albeit potentially with lower predictive accuracy. Prospective implementation studies and cost-effectiveness analyses are warranted to evaluate the added value of the metabolomic markers and the feasibility of large-scale adoption. Nonetheless, the nomogram presented in this study provides an immediately usable, paper-based tool for risk estimation, facilitating stepwise translation into clinical practice.

This study has several limitations. First, it is a single-center investigation. Although a rigorous training-validation set split and internal validation were performed, the model’s external generalizability requires future validation using multi-center, independent prospective cohort data to assess its performance across diverse populations and healthcare settings. Second, the prediction model was constructed solely based on baseline data from a single time point in the first trimester and does not account for the changes in these indicators throughout pregnancy. Future research could explore the incorporation of longitudinal prenatal monitoring data, utilizing time-series analysis or more sophisticated models to capture risk trajectories across gestation, thereby enabling more accurate risk prediction. Finally, while this study primarily focused on GDM prediction, the association between these early indicators and long-term offspring health outcomes (e.g., birth weight and future metabolic risk) warrants further investigation to extend the clinical value chain of the prediction model.

In conclusion, the combination of indicators highlighted by the model offers an integrative perspective on the early pathophysiology of GDM, encompassing glucose metabolism, lipid metabolism, inflammation, placental function, and amino acid metabolism. Future studies should focus on validating the model’s efficacy in broader populations and investigating whether its integration with clinical interventions can ultimately improve maternal and neonatal outcomes, thereby closing the loop from risk prediction to effective intervention.

## Data Availability

The original contributions presented in the study are included in the article/supplementary material, further inquiries can be directed to the corresponding author/s.
