# Supplementary material for: Development and validation of a model integrating clinical and metabolomic markers for gestational diabetes mellitus prediction
Source: Front Med (Lausanne). 2026 Jun 17;13:1806848. doi: 10.3389/fmed.2026.1806848 (PMC13318858; doi:10.3389/fmed.2026.1806848)
Supplement: Supplementary file 1 [file Table_1.docx]

Q11

**Supplemental Table 1.** Variable Assignment Table

| Variable | Meaning | Assignment |
| --- | --- | --- |
| X1 | Pre-pregnancy BMI | Continuous variable |
| X2 | FBG in First Trimester | Continuous variable |
| X3 | Triglycerides in First Trimester | Continuous variable |
| X4 | CRP in First Trimester | Continuous variable |
| X5 | PAPP-A | Continuous variable |
| X6 | Total Branched-Chain Amino Acid Score | Continuous variable |
| X7 | 1,5-Anhydroglucitol | Continuous variable |
| Y | GDM | (1=GDM group，0=Non-GDM group) |
